# Increasing Metagenomic Resolution of Microbiome Interactions Through Functional Phylogenomics and Bacterial Sub-Communities

**DOI:** 10.3389/fgene.2016.00004

**Published:** 2016-02-10

**Authors:** Angélica Cibrián-Jaramillo, Francisco Barona-Gómez

**Affiliations:** Laboratorio Nacional de Genómica para la Biodiversidad (Langebio), Unidad de Genómica Avanzada, Centro de Investigación y de Estudios Avanzados del Instituto Politécnico Nacional (Cinvestav)Irapuato, Mexico

**Keywords:** microbiome, metagenomics, functional phylogenomics, co-culture, bacterial interactions, specialized metabolites

## Abstract

The genomic composition of the microbiome and its relationship with the environment is an exciting open question in biology. Metagenomics is a useful tool in the discovery of previously unknown taxa, but its use to understand the functional and ecological capacities of the microbiome is limited until taxonomy and function are understood in the context of the community. We suggest that this can be achieved using a combined functional phylogenomics and co-culture-based experimental strategy that can increase our capacity to measure sub-community interactions. Functional phylogenomics can identify and partition the genome such that hidden gene functions and gene clusters with unique evolutionary signals are revealed. We can test these phylogenomic predictions using an experimental model based on sub-community populations that represent a subset of the diversity directly obtained from environmental samples. These populations increase the detection of mechanisms that drive functional forces in the assembly of the microbiome, in particular the role of metabolites from key taxa in community interactions. Our combined approach leverages the potential of metagenomics to address biological questions from ecological systems.

## Introduction

Biological understanding of the microbiome, defined as the set of microorganisms and their genomes in a particular environment (Boon et al., 2014), is one of the most exciting frontiers in science. High-throughput sequencing of single markers (16S rRNA gene) and shotgun metagenomics are now commonly used to describe the microbiome, revealing the presence of novel taxa (Wu et al., 2011; Lok, 2015); and increasing our understanding of the intimate interactions between symbiotic microbiomes and their hosts, in what has been termed the holobiont (Wilson et al., 2014). In the holobiont, the microbiome functions as an extended pheno/genotype of the host (Bordenstein and Theis, 2015), their rapid generation times enhancing the host’s ability to adapt quickly, and likely providing adaptive metabolites or the enzymatic machinery to produce them (Rosenberg et al., 2010). Indeed, the microbiome and its host likely act as a single evolutionary unit, of which we know very little of.

A major challenge to understand the host–microbiome interaction and the holobiont with its environment, is to define the intersection between taxonomic and functional diversity of the microbiome. That is, who are the members of the microbial community, who is merely co-existing, who is interacting and how; and how this could impact the evolution of the holobiont. To date, most metagenomic approaches on their own, lack sequencing depth to identify taxonomic groups, assemble and annotate functional pathways, but most importantly, recover ecologically key taxa and their associated metabolites that could identify bacterial interactions within the community.

**FIGURE 1:**
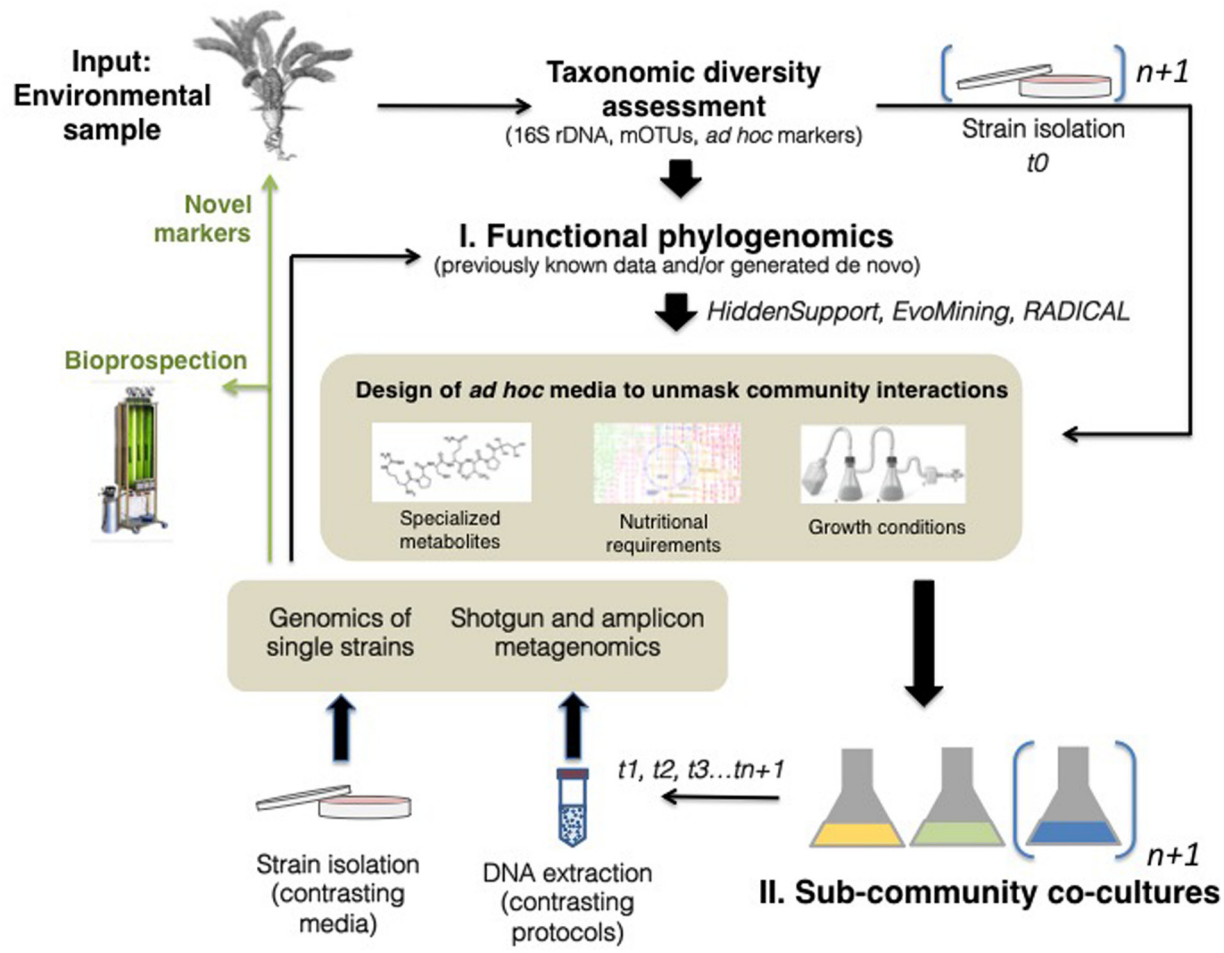
**A combined phylogenomics and experimental approach to enhance measurement of bacterial interactions within the microbiome.** Starting with an environmental sample, it would be possible to describe its taxonomic diversity, and use this information as well as published information (e.g. related taxa), to carry out phylogenomic analyses such as Hidden Support, EvoMining or RADICAL (see text for details). The main outcome (bold arrow) of the Functional Phylogenomics step is to identify genes with unique evolutionary and functional signatures that can be used to design *ad hoc* media co-cultures, in the form of information related to specialized metabolites, nutritional requirements or growth conditions. Different sub-community co-cultures can then be designed, and these could be measured at different time points, from days to years (t1, t2, t3…). Contrasting DNA extraction techniques from co-cultures would help reveal various types of taxonomic groups, measured with metagenomic strategies. Likewise, strain isolation could be carried out to sequence individual genomes in-depth. The main outcome of the sub-community cultures is information (genomes, genes) that can be annotated in-depth in the context of a simplified but natural community. This information can be used to re-design the *ad hoc* media into new sub-communities that target specific groups, used as part of biosprospecting strategies, or as novel markers that can be used to validate the presence of the community members in the original environmental sample.

We are mainly interested in increasing our ability to provide evolutionary and functionally relevant information of the microbiome community. We suggest a combined use of phylogenomic predictions and sub-community co-cultures in which biodiversity and their interactions are better understood. In particular, we are concerned with how to simplify the search for genetic information of functional importance from metagenomes, and how to simplify the composition of the microbiome enough that functional annotation can be interpreted in the context of bacteria-bacteria and bacteria-host interactions. One product of this combined approach is to generate functional information (e.g., gene clusters) that is presumed to have ecological and evolutionary relevance, and can therefore be used to validate biological hypotheses, by characterizing them back in an environmental sample or by downstream experimentation (**Figure 1**).

We propose two complementary strategies to achieve the goal of identifying and using functional and evolutionary meaningful information to understand the microbiome community. First, the use of phylogenomic tools to identify gene clusters or gene families with a distinct evolutionary pattern that can be used to infer taxonomic patterns and functional roles in the bacterial community. The main advantage of this computational approach is that these candidate genes constitute a reduced universe of evolutionary hypothesis based on function, which can be tested experimentally and/or with down-stream bioinformatics, rather than experimenting more broadly in the entire genome or metagenomic dataset. It differs from current efforts to determine phylogenetic diversity focused on 16S rRNA gene or markers with phylogenetic signal, such as metagenomic operational taxonomic units (mOTUs) (Sunagawa et al., 2013); or profiling methods based on many markers (Nguyen et al., 2014). It complements methods based in characterizing metacommunity of genes with some functional relevance (Boon et al., 2014), and methods that attempt to identify functional genes in the context of partitioning bacterial genomes into pan/shell genomes (Shi and Falkowski, 2008; van Tonder et al., 2014) or using information theory to quantify the degree of conflict or incongruence calculated from different types of data (Salichos et al., 2014), although we argue that a finer partition beyond a core and pangenome is required to be able to identify functional genes, perhaps more similar to GO-based phylogenetic classifications (Chai et al., 2014). It would also be possible to use metatranscriptomic profiling to help narrow down gene candidates of adaptive value based on their differential expression (Franzosa et al., 2014).

Second, we suggest that it is possible to sample sub-communities of the original environmental sample using co-culture strategies, under the assumption that functional interactions mediated by genes and their products will be easier to detect in more simple, pre-conceived functionally driven culture conditions. A distinctive advantage of the co-cultures over traditional microcosms whose reproducibility has been recently questioned (see e.g., Langenheder et al., 2006; Pagaling et al., 2014), is that interactions within a complex system may be better resolved by dissecting it onto sub-communities based on functional concepts. Functionally important genes identified with phylogenomic strategies can be used to guide the design of the co-cultures themselves, in the form of metabolites that can drive community interactions, nutrients required or even biological growth conditions (**Figure 1**). Co-cultures in themselves enable testing of directed hypotheses, and can generate data that can be used as a source of novel *ad hoc* markers, which can be validated in the original biological sample and used to re-design new co-cultures that enhance our ability to understand bacteria interactions (**Figure 1**).

The implications of knowing key functional players and their interactions in the microbiome enlightens evolutionary biology, but can also help solve two major issues in bioprospecting of secondary or specialized metabolites (Charlop-Powers et al., 2015; Ling et al., 2015), commonly known as secondary metabolites or natural products. That is, the discovery of genes encoding for novel enzymes; and ‘turning on’ biosynthetic genes directing the synthesis of specialized metabolites in ways that can be adopted by synthetic biology approaches (Luo et al., 2015; **Figure 1**), facilitating the transition from pattern descriptions into deciphering mechanisms at different levels (Waldor et al., 2015).

## Phylogenomics to Detect and Classify Genes of Functional Importance in the Microbiome

We describe three strategies aimed at identifying genes that have evolutionary and ecological relevance, and identifying hidden functional diversity within metagenomes in the context of a phylogeny, based on **(a)** revealing hidden support of genes within a concatenated alignment, as the alignment matrix resulting from the concatenation of all gene/protein partitions that are orthologous among the targeted genomes; **(b)** measuring the emergence of specialized metabolism in a topology to discover hidden chemical diversity; and **(c)** identifying clades and gene categories with incongruent evolutionary signals that suggest horizontal gene transfer (HGT), genome streamlining, or unique evolutionary trajectories (e.g., Shi and Falkowski, 2008). In combination, these phylogenomic methods can reveal ‘functional units’ that can be identified and classified, also helping to resolve species delimitations and their phylogeny, although this is not our main goal.

### (a) Hidden Support

Work by one of the co-authors (Cibrián-Jaramillo et al., 2010; Lee et al., 2011) previously developed a phylogenomic approach to identify genes of functional importance in plants. We suggest that this approach can be transferred to bacterial metagenomes and genomes to identify genes that have different functional roles, and biological processes putatively involved in species diversification. In this method, the authors provide a way to study the behavior of genes used to reconstruct a phylogeny through analysis of their effect on tree topology and branch support (Cibrián-Jaramillo et al., 2010). This approach is fundamentally different than classical phylogenetic analysis because the search for both orthologs and candidate genes is conducted with a phylogeny. If one assumes that the tree obtained from concatenated analysis using these orthologs best represents the evolutionary history of the taxa involved, then phylogenetic incongruence between a partitioned functional class of genes and the organismal phylogeny would suggest that the partition (gene) has experienced a unique evolutionary history relative to the organisms. These genes are essentially a set of hypotheses of functions and evolutionary mechanisms that can be validated experimentally.

### (b) EvoMining

**FIGURE 2:**
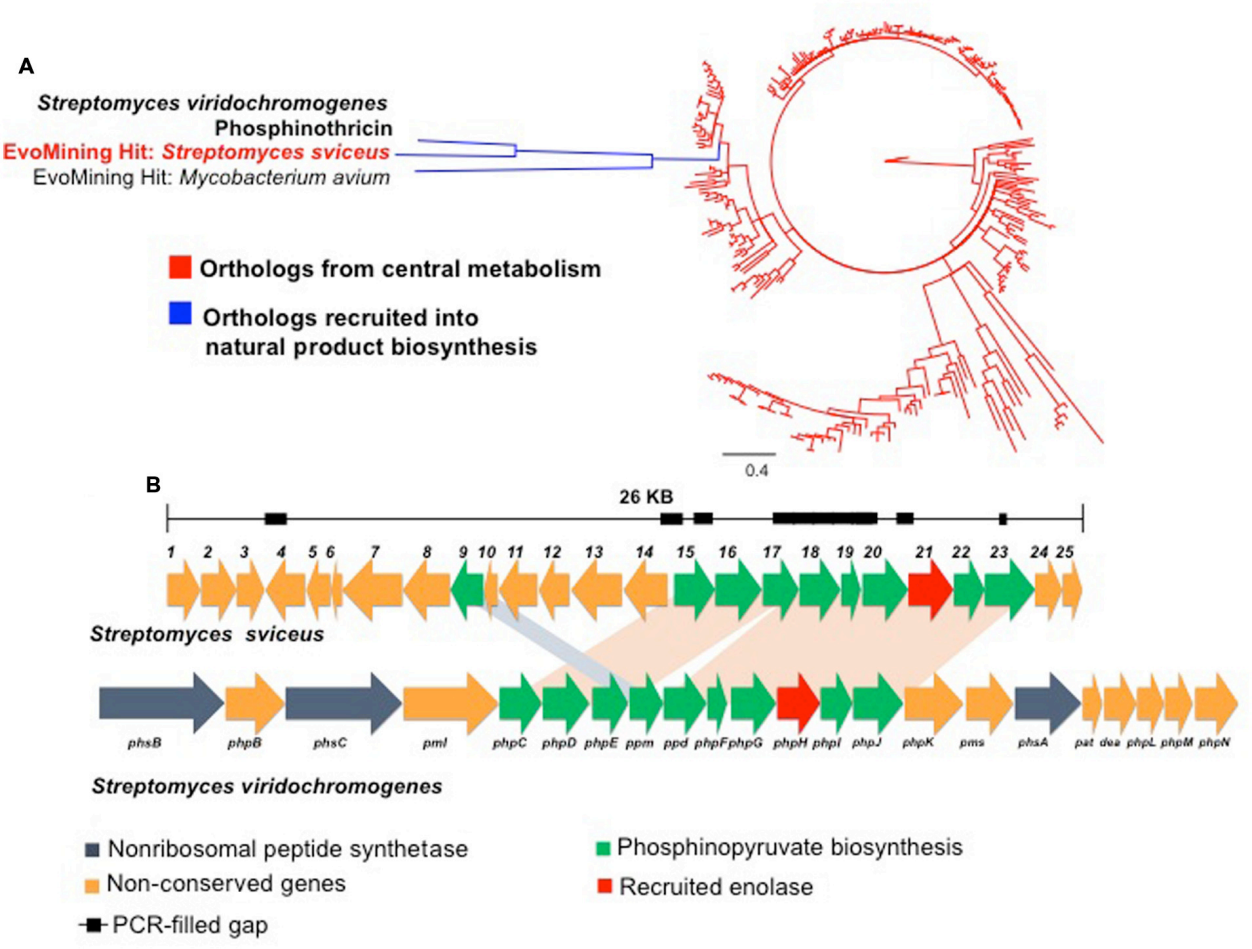
**EvoMining example. (A)** Phylogenetic reconstruction of enolases extracted from an *Actinobacteria* genome database. After manual annotation, homologs believed to be involved in glycolysis were labeled in red. The clade predicted to include enolase homologs involved in natural product biosynthesis, including that from *S. sviceus* (EvoMining hit), is shown in blue. **(B)** Structural organization of the natural product biosynthetic gene cluster identified using EvoMining on the genome of *S. sviceus* (top). After manual closure of this locus after sequencing of PCR products (black boxes) the emerging cluster was annotated and compared with the organization of the biosynthetic gene cluster known to direct the synthesis of phosphinotricin in *S. viridochromogenes* (bottom) (Blodgett et al., 2015).

Cruz-Morales et al.^1^ developed a functional phylogenomics platform to identify expanded, repurposed enzyme families, which have been recruited from central metabolism into the context of specialized metabolism. EvoMining uses single-gene topologies to reveal characteristic phylogenetic signatures of rapid evolution that can be further analyzed in detail with the construction of hidden Markov models (HMM) profiles. These mechanisms leave a phylogenetic signature that can be measured in a topology by means of both clade formation and sequence divergence. Functional annotation of the phylogenetic tree, on the basis of genome-mining approaches (e.g., antiSMASH; Medema et al., 2011), provides validation and insights for visual inspection of the data. As this approach does not rely on sequence similarity searches of previously identified enzymes, but rather on recapitulation of an enzyme evolutionary process, it is less sensitive to missing data, which is relevant for low quality draft genomes (e.g., metagenomes). Subsequently, functional specificities of identified enzymes can be explored (Noda-García et al., 2015). An example of how EvoMining can lead to hidden signals in the context of metagenomics is provided in **Figure 2**. We show an EvoMining hit in the enolase enzyme family found in the highly fragmented genome of *Streptomyces sviceus* (1 scaffold of 9 Mbp with 552 gaps and 8X coverage, GI: 297196766). Its contig containing a recruited enolase (GI: 297146550) typically involved in glycolysis, had 6 gaps including missing sequence at its 5′ end. After closing gaps (PCR), the complete sequences for several phosphonate-related enzymes, namely, alcohol dehydrogenase (*phpC*), phosphonopyruvate decarboxylase (*ppd*), nicotinamide mononucleotide adenyl transferase (*phpF*), carboxy-phosphonoenolpyruvate synthase (*phpH*, EvoMining hit), and aldehyde dehydrogenase (*phpJ*), could be annotated. Further sequence analysis suggested that indeed this locus encodes for a putative phosphinate biosynthetic gene cluster related to the phosphonic tripeptide phosphinothricin. This was recently confirmed by in-depth comparative genomics analyses (Blodgett et al., 2015; **Figure 2**).

### (c) RADICAL

The main goal of RADICAL is to reveal aspects of phylogenetic behavior otherwise not evident in individual gene trees or ‘total evidence’ trees (Narechania et al., 2012). RADICAL sequentially concatenates randomly chosen gene partitions starting with a single-gene partition and ending with the entire genomic data set, with a tree built for every successive addition; until a library of trees is generated representing a large variety of differently sized random gene partitions. RADICAL analyses in cyanobacteria using gene subgroups confirmed that there are elevated levels of incongruence for genes involved in metabolism, but also showed that the distinction of ‘core’ and ‘pan/shell’ genome is not clear-cut. Furthermore, metabolism genes were the only functional class of genes supporting the monophyly of *Prochlorococcus*, suggesting that if indeed metabolism genes are affected by HGT more than other types of genes, there is virtually no support for a cohesive grouping of species within this clade (Narechania et al., 2012). Support for monophyletic groups can be used to identify which genes are congruent with species delimitations based on 16S rRNA genes or highly conserved markers, including mOTUs (Sunagawa et al., 2013) to distinguish phylogenetic history from functions due to community interactions.

Hidden Support and RADICAL are based on concatenation and topological congruence (Rokas et al., 2003; Gatesy, 2005). In essence, metagenomic data would be partitioned into evolutionary units independent of a core and pan-genome category, to reveal any genes that have different phylogenetic histories, but also those that affect the topology as a result of biased, missing data, or errors in annotation. The partition of metagenomes into evolutionary units is not a trivial task but it would be possible to deconvolute genes and identify orthologs from metagenomic data using binning methods that recover discrete units from metagenomic sequences (Mackelprang et al., 2011; Sharon et al., 2013; Alneberg et al., 2014); or by calculating gene abundances across various samples and correlating it with taxonomic distribution, such that we have taxon-specific profiles (Carr et al., 2013). A phylogeny-driven Bayesian test for the presence of an organism in a sample could also be done (Darling et al., 2014); or a combined strategy of ortholog clustering (e.g., OrthoMCL) followed by a phylogeny to identify and single out orthologs from paralogs (Chiu et al., 2006). Genes resulting from any of these methods of metagenomic extraction can be used to construct the trees required for Hidden Support and RADICAL.

In sum for this section, phylogenomic data would enable a *top–bottom* strategy in which we could predict metabolites from metagenomic sequences that are community interactors or mediators. We would be able to validate the role of keystone taxa through annotated gene clusters in bacterial metacommunities, in particular those that are essential for adaptive bacterial phenotypes or function as community interactors such as persister variants in antimicrobial resistance (Amato et al., 2014) or siderophore biosynthesis (D’Onofrio et al., 2010). It would be possible to develop markers (e.g., oligos based on the sequence of the identified functionally important genes) that can be amplified in an environmental sample or in an experimental culture as described below, to test their presence, distribution, and biological role in the microbiome (**Figure 1**).

## Identifying and Testing the Role of Functional Interactors From Metagenomes

The main question in this section is how do we define the interactions within a community in the context of the microbiome? We adopt the definition of community as ‘multispecies assemblages, in which organisms live together in a contiguous environment and interact with each other’ (Konopka, 2009). Early views of the community in ecology imply a tight interaction among its members (Clements, 1916) perhaps akin to the modern idea of the metacommunity in which there is dynamic movement of genes through microbial lineages (Boon et al., 2014), but more importantly, metabolic dependencies between community members (Hug et al., 2012). Thus, a community can be hypothesized as metabolic interactions that can be tested in the context of the microbiome’s environment and/or its relationship with a host (i.e., holobiont).

The main technical issue, however, is to distinguish coexistence from a spectrum of interactions among the members of a set within a niche, viewed as ‘a subset of those environmental conditions which affect a particular organism, where the average absolute fitness of individuals in a population is greater than or equal to one’ (Kearney, 2006). Our main premise is that microbiome interactions can be described by experimentally testing and manipulating their role in the community assembly processes as it is done in eukaryote ecology (Fayle et al., 2015). In the bacterial microbiome, metabolites and the genes that underlie their presence and variation could be used as a proxy for interactions, including the identification of ecologically key organisms (Mills et al., 1993).

To achieve this we propose the use of functionally driven sub-community co-cultures, which are preceded by synthetic co-cultures typically involving arrays of isolates in the format of two strains at the time. In the best-case scenario, the latter may come from the same sample but not necessarily known to interact. Yet, co-cultures are increasingly used to understand general mechanisms by which bacteria may interact among themselves and with eukaryotic cells by means of metabolic exchange (Traxler and Kolter, 2015). The chemotypes sustaining such interactions can be assessed by high-resolution analytical approaches, such as imagining mass spectrometry directly from Petri dishes (Hoefler et al., 2012) and nuclear magnetic resonance (Wu et al., 2015). Even shallow sequencing coverage of these sub-populations would be enough to reach resolution of species diversity and the possibility to measure novel specialized metabolites directly in the context of the sub-community. In contrast with metagenomes obtained directly from environmental sampling, we do not aim to have thorough ecological representation. *Ad hoc* co-culture conditions are simply a proxy for the mechanistic niche in which we increase metagenomic resolution of a simplified metabolic niche.

Co-cultures of sub-communities starting from inoculum directly obtained from environmental samples, can be designed in various ways. For example, using substrates that enhance diversity which are initially enriched for a particular bacterial genus or species suspected or shown to be prevalent in environmental samples. Additionally, sub-communities can be recovered from media that are as similar as possible to the original hosts or sites (Ling et al., 2015) or based on genome-scale metabolic network reconstructions from phylogenomic data, leading to nutritional requirements of key taxa (Barona-Gómez et al., 2012; **Figure 1**).

Thus, co-cultures which may be followed through various time series (days or years) would enable growth of typically rare species (Pagaling et al., 2014); genomic lineages with functional traits that are not easily recovered; or species that sustain metabolic exchanges with other bacterial symbionts. This could unmask genomic-lineages undergoing evolutionary processes, for example those driven by differential gene gain-and-loss. By genome sequencing of those strains, which may be recovered from the co-cultures, the resolution of the microbiome using metagenomics would be increased. In contrast with synthetic populations (Hoefler et al., 2012), these sub-communities represent an experimental platform to reduce ecological complexity including at least some of the original members of the biological sample. They represent a complement to microcosms in that reproducibility and predictability, and thus biological resolution, is expected to increase in the co-cultures. Eventually we would have enough resolution to detect inactivated genes and pathway degeneration, as well as appearance of novel pathways, in certain niches that result from local adaptation (Hittinger et al., 2004), and as sequencing depth increases, metabolic networks in the context of the community could be reconstructed (Klitgord and Segrè, 2011).

Metagenomic data from sub-community populations also provides the possibility of interpreting species distributions with community ecology methods (reviewed in Gerhold et al., 2015). As proposed to date, these methods are oversimplified and are biased due to inaccurate taxonomic annotations of intrinsically low-resolution data (Gerhold et al., 2015). In sub-community co-cultures, increased accuracy of functional annotation would enable testing of trait distribution to infer the role of competition or facilitative interactions for example, increasing our understanding of the ecological processes in the microbiome.

Finally, metagenomic sequencing of co-cultures should be coupled with sampling strategies that enable explicit hypothesis of local adaptation and increase the likelihood of identifying genes and their metabolites with specialized functions in the community. For instance, sampling with biological replicates along environmental gradients, contrasting habitats, or within specialized organs or structures (gut, nodules, specialized roots, etc) would be examples of targets that could increase ecological resolution in interpretation of the microbiome interactions.

## Conclusion

To increase our understanding of the microbiome’s evolutionary processes, and in particular of the interactions of the microbial community and its environment and hosts, phylogenomics can be used to identify functionally important genes, and co-cultures can be used to approximate sub-community interactions. In combination, our understanding of the microbiome community taxa composition and their interactions will be greatly enhanced.

## Author Contributions

AC-J and FB-G both equally conceived the ideas for this manuscript, wrote the text, and revised it critically for intellectual content.

## Conflict of Interest Statement

The authors declare that the research was conducted in the absence of any commercial or financial relationships that could be construed as a potential conflict of interest.

## Footnotes

1 http://www.biorxiv.org/content/early/2015/06/08/020503.abstract
